# Is proton beam therapy always better than photon irradiation? Lessons from two cases

**DOI:** 10.1002/jmrs.773

**Published:** 2024-03-20

**Authors:** Michelle P Li, Adam Yeo, Roshini Gunewardena, Gabrielle Drum, Kirsty Wiltshire, Claire Phillips, Joseph Sia, Greg Wheeler, Lisa Hall

**Affiliations:** ^1^ Peter MacCallum Cancer Centre Melbourne Victoria Australia; ^2^ Sir Peter MacCallum Department of Oncology University of Melbourne Melbourne Victoria Australia; ^3^ School of Applied Science RMIT University Melbourne Victoria Australia

**Keywords:** Comparative treatment planning, ependymoma, optic pathway glioma, paediatric radiotherapy, proton beam therapy, proton therapy, protons, stereotactic radiotherapy

## Abstract

Proton beam therapy (PBT) is increasingly used to treat cancers, especially in the paediatric and adolescent and young adult (AYA) population. As PBT becomes more accessible, determining when PBT should be used instead of photon irradiation can be difficult. There is a need to balance patient, tumour and treatment factors when making this decision. Comparing the dosimetry between these two modalities plays an important role in this process. PBT can reduce low to intermediate doses to organs at risk (OAR), but photon irradiation has its dosimetric advantages. We present two cases with brain tumours, one paediatric and one AYA, in which treatment plan comparison between photons and protons showed dosimetric advantages of photon irradiation. The first case was an 18‐month‐old child diagnosed with posterior fossa ependymoma requiring adjuvant radiotherapy. Photon irradiation using volumetric modulated arc therapy (VMAT) had lower doses to the hippocampi but higher doses to the pituitary gland. The second case was a 21‐year‐old with an optic pathway glioma. There was better sparing of the critical optic structures and pituitary gland using fractionated stereotactic radiation therapy over PBT. The dosimetric advantages of photon irradiation over PBT have been demonstrated in these cases. This highlights the role of proton‐to‐photon comparative treatment planning to better understand which patients might benefit from photon irradiation versus PBT.

## Graphical abstract

This report describes two paediatric/adolescent and young adult (AYA) brain tumour cases demonstrating the dosimetric advantages of photon irradiation over proton beam therapy. This highlights the role of proton‐to‐photon comparative treatment planning to better understand which patients might benefit from photon irradiation versus proton beam therapy.

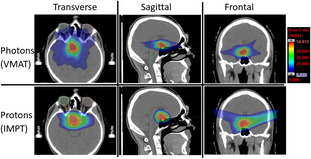

## Introduction

Proton beam therapy (PBT) has physical properties that enable reduced low to intermediate dose to normal tissue outside of the tumour volume.^1^ There is ongoing research to determine if PBT results in reduced acute and late toxicities and better cancer outcomes compared with photon irradiation. As the number of PBT facilities increases worldwide, this treatment is becoming more accessible to all cancer patients. However, treatment remains more expensive and resource‐intensive compared with photon irradiation. It is important to develop a method to select which patients would benefit most from PBT compared with photon irradiation.^2^

In many countries, PBT for paediatric and AYA patients with cancer is becoming the ‘standard of care’. This model assumes that PBT is superior to photon irradiation and this patient population would benefit the most from this treatment. In Australia, a patient's clinical team must submit proton‐to‐photon comparative treatment plans to support their Medical Treatment Overseas Program (MTOP) application to receive financial support from the Australian Federal Government for PBT overseas. In Australia, dosimetry is one of the multiple factors including cancer prognosis, the urgency of treatment and patient's family and support network, which help determine the recommended radiation treatment.^3^ It is anticipated that proton‐photon comparative treatment planning will still be required when Australia's first PBT facility opens in Adelaide in 2025.^4^

Here we present two patients with brain tumours who had a photon and an in‐house proton plan generated to help decide whether a MTOP application for overseas PBT should be submitted.

## Case 1

The 18‐month‐old patient presented with a month history of vomiting. Magnetic resonance imaging (MRI) indicated a large posterior fossa tumour within the fourth ventricle measuring 38 × 35 × 70 mm. There was the significant mass effect on the brainstem and cerebellum causing marked obstructive hydrocephalus. There was no evidence of spinal disease. The patient underwent a posterior fossa craniotomy and gross total resection of the tumour. Post‐operative imaging did not show any evidence of residual disease. Histopathology showed an ependymoma (World Health Organisation (WHO) grade 2) with immunonegativity of H3K27me3, consistent with posterior fossa group A ependymoma. CSF sampling completed 2 weeks after surgery was negative for malignancy.

The patient's case was discussed at a multidisciplinary team (MDT) meeting and recommended for radiation therapy. Due to the patient's young age and the location of the tumour, proton and photon plans were generated to help determine which treatment modality to use.

The patient completed a planning CT scan acquired with 120 kV and 2 mm slice thickness (Philips Brilliance Big Bore RT) under general anaesthesia according to the paediatric scanning protocol. The patient was positioned supine on a FreedomX board, immobilised with a personalised thermoplastic mask, Silverman headrest (CDR Systems, Calgary, Canada) and a paediatric moldcare pillow (Bionix Radiation Therapy, Toledo, Ohio) was created. The gross tumour volume (GTV) was delineated based on the tumour bed seen in the post‐operative MRI scan. An isotropic margin of 10 mm around the GTV was delineated and then cropped to anatomical boundaries to create the clinical target volume (CTV). For the photon plan, an isotropic margin of 3 mm was created around the CTV to create a planned target volume (PTV). The prescribed dose to the PTV was 54 Gy in 30 fractions and the plan was optimised for target volume coverage as per the ICRU83 guidelines.^5^ In PBT planning, the PTV is not created because coverage of the CTV under all uncertainty scenarios is considered equivalent to photon PTV coverage. The brainstem, optic structures, temporal lobes, hippocampi, pituitary gland and cochlea were delineated as organs at risk (OAR).

The photon plan was generated using Eclipse™ treatment planning system (TPS) V16.1 (Varian Medical Systems, Palo Alto, California, USA). The volumetric modulated arc therapy (VMAT) plan consisted of two full coplanar arcs and one partial non‐coplanar arc, entering superiorly to avoid entering and exiting through critical OAR.

The proton plan was generated using the Varian ProBeam model data on Eclipse™ TPS V16.1. The Varian ProBeam model was shared by Scripps Proton Therapy Center (San Diego, CA) for Eclipse proton user commissioning. Beam measurement data for commissioning included integrated depth dose curves with RBE1.1 conversion and beam spots. Both were measured every 5 MeV between 70 MeV and 244 MeV. The plan utilised intensity modulation proton therapy (IMPT) using a spot scanning technique and inverse planned optimisation. Multiple beam arrangements were trialled for the proton plan, both single‐field optimised (SFO) and multi‐field optimised (MFO). The beam models used a constant dose scaling factor of 10% to apply a fixed relative biological effectiveness (RBE) of 1.1.^6^ The final plan consisted of three beams, two posterior obliques and a posterior field. A 50 mm range shifter was utilised to ensure full dose coverage where the CTV was close to the skin surface. No other beam‐modifying devices were used. A robust target volume was created for each beam to facilitate spot placement and dose manipulation where required. Robust optimisation was performed, with parameters considering a 3.5% CT curve error and 3 mm geometric uncertainties in all three directions. The nominal plan was reviewed and 12 uncertainty scenarios were created to further evaluate the plan, using the previously described robust optimisation parameters (3.5%/3 mm).

**Figure 1:**
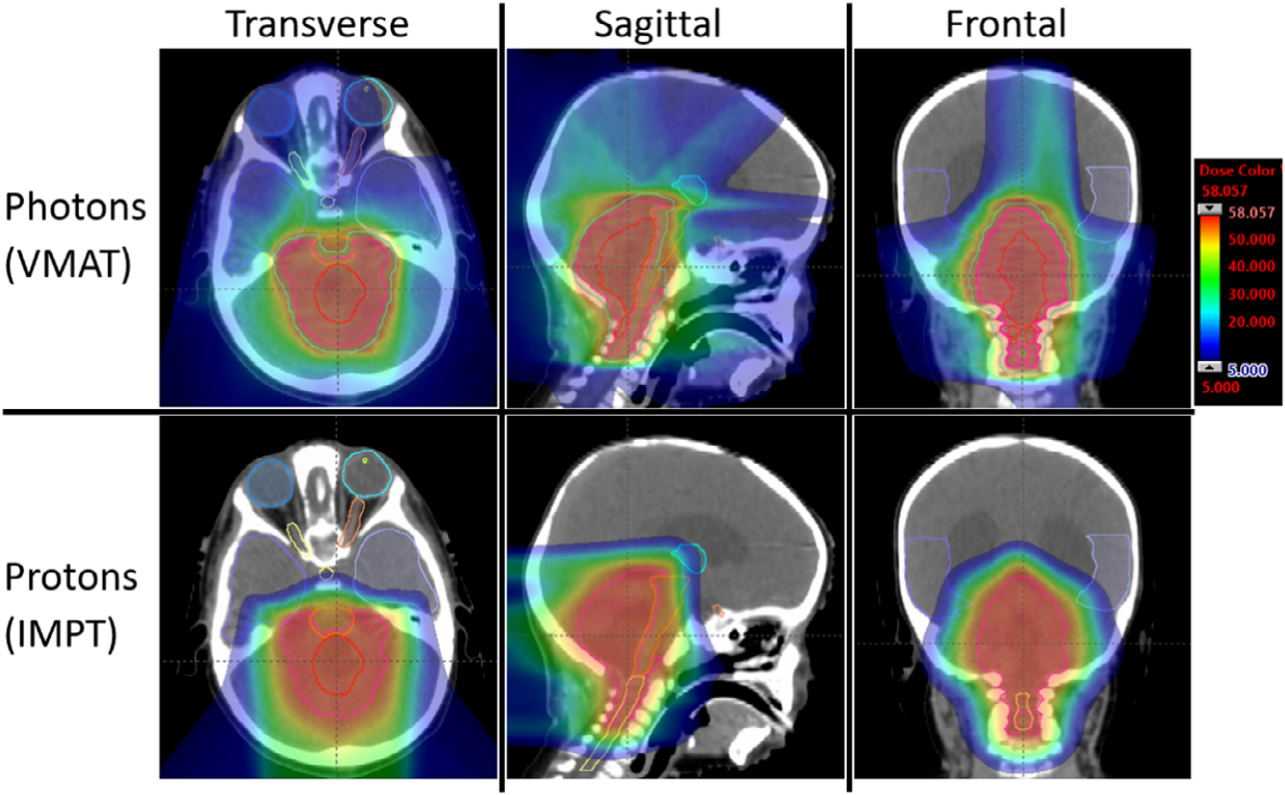
A comparison of dosimetry for Case 1 between Photon‐VMAT (top row) and IMPT on three orthogonal planes, overlaid structures include targets as well as organs‐at‐risk, e.g. brainstem, spinal cord, temporal lobes, thalamus, eyes, lens and optic nerves. Dose colour wash ranges from 5 Gy to 58 Gy (max dose 107.4%). For the IMPT plan, couch angle was 0° for all three fields. Field 1 gantry angle was 140°, Field 2 gantry angle was 180° and Field 3 gantry angle was 220°.

**Figure 2:**
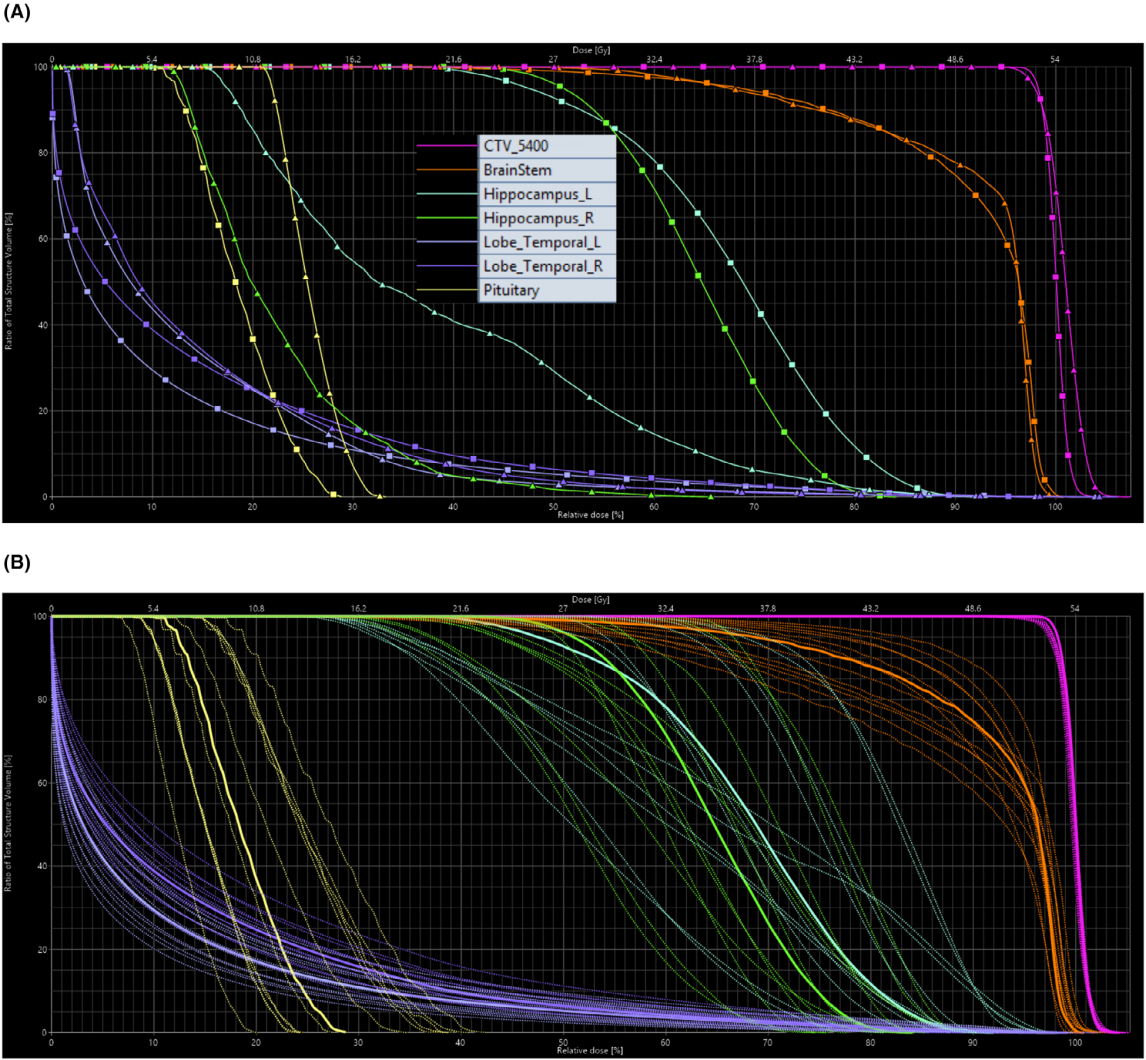
Dose‐volume histograms for Case 1: (A) nominal DVH comparison between Photons (Δ) vs Protons (□); (B) proton robust plan evaluation for the proton plan – a band of 12 DVH curves with uncertainty parameters of ±3.5% calibration curve error and ±3.0 mm of geometric error in all six directions. DVH axes show relative volume in % (y‐axis), relative dose in % (bottom x‐axis) and absolute dose in Gy (top x‐axis). Structures include CTV and organs‐at‐risk such as brainstem, hippocampi, temporal lobes and pituitary.

Figure 1 shows a comparison of dose distribution between photon and proton plans. Figure 2A depicts a DVH comparison of tumour coverage and dose to OAR between the photon and proton plans. Figure 2B illustrates robust evaluation for the proton plan with the aforementioned uncertainty scenarios where small volume OARs show large variation with the given uncertainty scenarios as expected. A comparison of tumour coverage and dose to OAR between the proton and photon plans is summarised in Table 1.

**Table 1:** Dose‐volume metrics scores for Case 1.

Structure	Dose‐volume metrics	Objectives	Proton‐IMPT	Photon‐VMAT	Difference (Gy) (IMPT‐VMAT)
PTV_5400	D98%	≥51.3 Gy	50.3^a^	51.8^a^	−1.5^a^
CTV_5400	D98%	≥51.3 Gy	52.8	52.4	0.4
Brainstem	D0.03 cc	≤54.0 Gy	54.3	54.1	0.2
Optic_Chiasm	D0.03 cc	≤54.0 Gy	11.4	21.4	−10.0
OpticNrv_L	D0.03cc	≤50.0 Gy	4.2	18.5	−14.3
OpticNrv_R	D0.03cc	≤50.0 Gy	6.1	24.2	−18.1
Pituitary	D0.03cc	≤15.0 Gy	15.5	17.9	−2.4
Hypothalamus	D0.03cc	≤15.0 Gy	29.5	25.6	3.9
Cochlea_L	Mean Gy	<35.0 Gy	29.7	29.5	0.2
Cochlea_R	Mean Gy	<35.0 Gy	30.3	29.4	0.9
Lobe_Temporal_L	Mean Gy	<10.0 Gy	5.8	7.3	−1.5
Lobe_Temporal_R	Mean Gy	<10.0 Gy	7.4	7.8	−0.4
Hippocampus_L	Mean Gy	<14.0 Gy	36.6	20.8	15.8
Hippocampus_R	Mean Gy	<14.0 Gy	34.8	12.2	22.6
Gradient Index^b^	(= V50%/V100%)		8.58	4.44	
Homogeneity Index^c^	(= D2%/D98%)		1.05	1.07	
Integral Dose	Gy cc		24,035.99	37,125.13	37,125.13

L, left; OpticNrv, optic nerve; R, right.

^a^ PTV coverage for the Proton plan is not applicable as CTV‐based robust optimisation is implemented but displayed for illustration purposes only. VMAT PTV D99% >95% of prescription dose ensures excellent robustness against setup error, which is confirmed by OBI kV‐kV pair for bony matching.

^b^ Gradient index is defined by the ratio of V50% to V100% of prescription dose.^7^

^c^ Homogeneity Index is defined by the ratio of near‐max (D2%) to near‐min (D98%).^8^

Coverage of the tumour volume was similar in both treatment plans. The dose to the hippocampi was reduced in the photon plan. The dose to the right temporal lobe and cochlea was comparable. However, the dose to the pituitary gland, optic structures and left temporal lobe was higher in the photon plan. Thus the photon plan could have a greater impact on endocrine dysfunction, whereas the proton plan could have a greater impact on neurocognitive dysfunction. The integral dose was lower by 35% in the proton plan compared with the photon plan. This translated into a 40% dose reduction in the normal brain in terms of V12 Gy, which was 23.1% (246.7 cc) in the proton plan, compared to 38% (405.4 cc) in the photon plan. Due to the patient's young age, the clinical team and the patient's parents favoured the photon plan over the proton plan to minimise dose to bilateral hippocampi.

## Case 2

This patient was initially diagnosed with a right optic pathway glioma at the age of eight presenting with visual loss and proptosis. MRI showed a 22 × 21 × 28 mm mass expanding and distorting the right optic nerve.

The patient underwent 12 doses of monthly carboplatin, which achieved control of the tumour. However, surveillance imaging indicated disease progression 4 years later, for which debulking surgery was performed. Histopathology confirmed a diagnosis of pilocytic astrocytoma. Subsequent MRI scans showed chiasmatic residual disease measuring 6 × 8 × 4 mm which was observed and remained stable until the age of 20 years old, when there was progression to 11 × 15 × 9 mm. The patient underwent 4 cycles of 3 weekly carboplatin but MRI indicated further growth to 17 × 14 × 9 mm involving the optic chiasm.

The patient's case was discussed in the neuro‐oncology MDT meeting. Options of biopsy to guide targeted systemic treatment where possible, upfront vinblastine chemotherapy or radiation therapy were recommended. The patient decided to proceed with radiation therapy. Due to the patient age and central tumour location, photon and proton plans were generated.

Given the small volume and its location, a stereotactic approach was adopted for CT simulation with 140 kV and 1 mm slice thickness (Philips Brilliance Big Bore RT) according to the neuro‐stereotactic protocol. The patient was positioned supine and immobilised with a stereotactic thermoplastic mask with reinforced areas around the forehead and chin (NL‐TEC PTY LTD, Willetton, Western Australia) compatible with the Brainlab robotic ExacTrac system (Brainlab AG, Munich, Germany). The use of this mask system allowed for tighter planning margins and greater accuracy in treatment delivery. GTV was delineated based on the diagnostic MRI. An isotropic margin of 1.0 mm was delineated around the GTV to create the CTV. For the photon plan, an additional margin of 0.5 mm clipped to anatomical boundaries was used for PTV. GTV volume was 1.9 cm^3^ and PTV volume was 3.7 cm^3^. The prescribed dose to the PTV was 50.4 Gy in 28 fractions and the plan was optimised for target volume coverage as per the ICRU83 guidelines.^5^

The photon plan was created using three volumetric modulated arcs; one full co‐planar arc and two non‐coplanar arcs of 200° in length each with ±45° of couch kicks. The treatment plan was designed to be compatible with stereotactic setup and delivery coupled with six degrees of freedom image‐guided radiation therapy (IGRT) corrections (0.5 mm/0.5° IGRT actional levels by ExacTrac), although the radiation prescription was not stereotactic. The proton plan was created using two lateral non‐coplanar fields angled slightly superiorly to better avoid the pituitary gland, positioned inferior to the target volume. No beam‐modifying devices were used. Alternative beam arrangements were attempted including the addition of a third beam, however, these changes led to a higher pituitary dose. The same TPS details and plan robustness evaluation technique were used as specified in Case 1.

**Figure 3:**
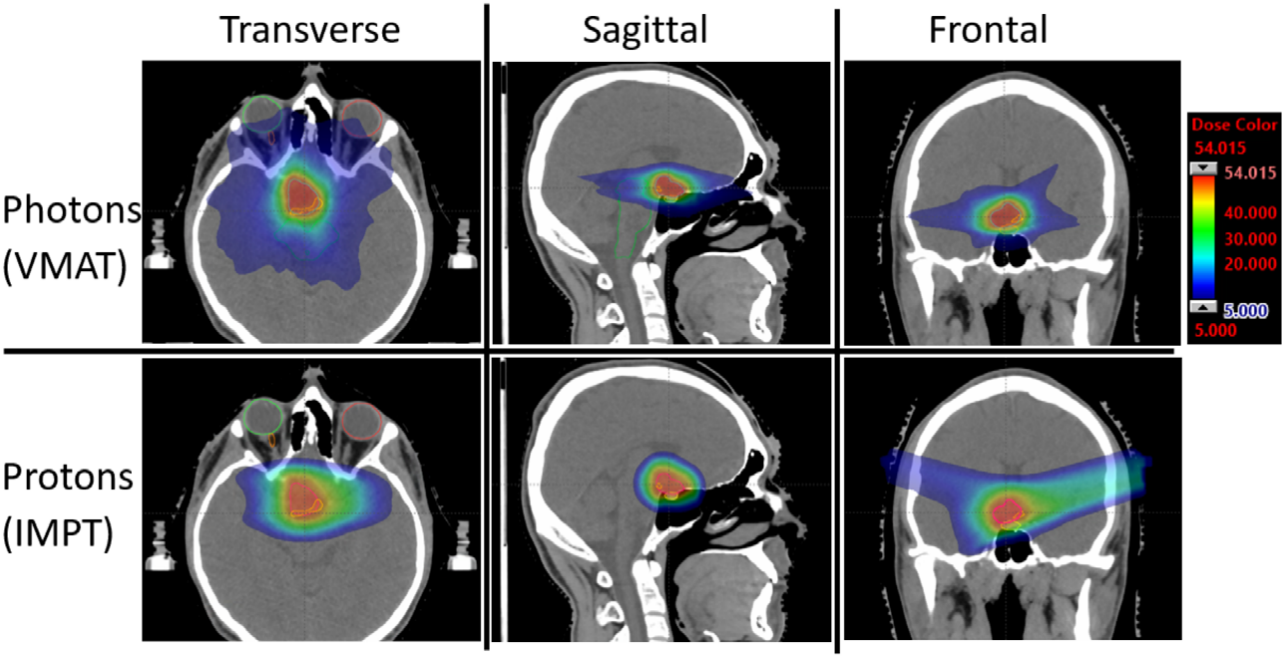
A comparison of dosimetry for Case 2 between VMAT (top row) and IMPT on three orthogonal planes, overlaid structures include targets as well as organs‐at‐risk, e.g. brainstem, spinal cord, temporal lobes, thalamus, eyes, lens, and optic nerves. Dose colour wash ranges from 5 Gy to 58 Gy (max dose 107.4%). For the IMPT plan, Field 1 gantry angle was 270° and couch angle was 17°, Field 2 gantry angle was 90° and couch angle was 343°.

**Figure 4:**
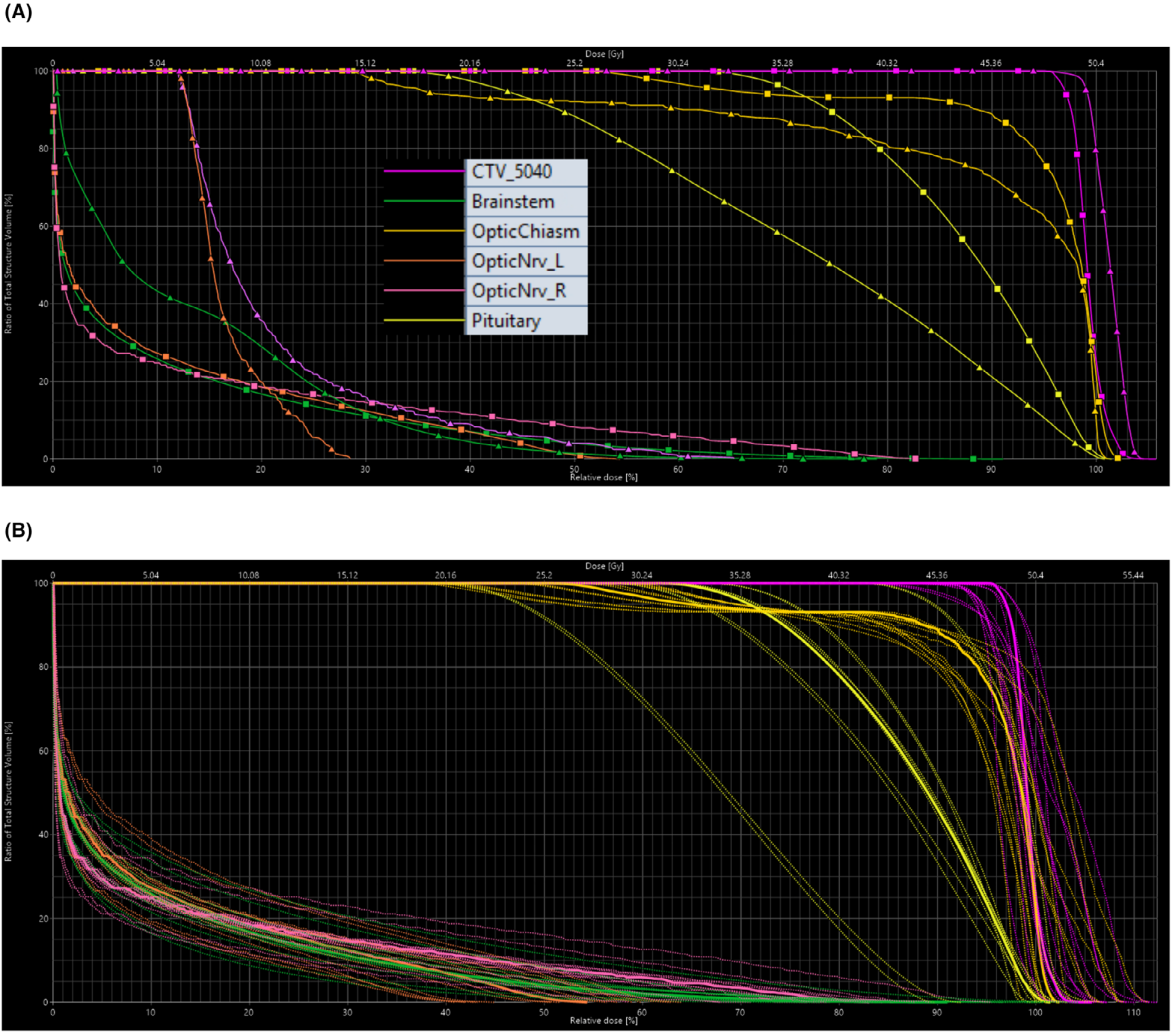
Dose‐volume histograms for Case 2: (A) nominal DVH comparison between Photons (Δ) vs Protons (□); (B) proton robust plan evaluation for the proton plan – a band of 12 DVH curves with uncertainty parameters of ±3.5% calibration curve error and ±3.0 mm of geometric error in all six directions. DVH axes show relative volume in % (y‐axis), relative dose in % (bottom x‐axis) and absolute dose in Gy (top x‐axis). Structures include CTV and organs‐at‐risk such as brainstem, optic chiasm, optic nerves and pituitary.

Figure 3 shows a comparison of dose distribution (ranging from global max dose to 5 Gy low dose‐bath) through three orthogonal planes between photon and proton plans. Figure 4A depicts a DVH comparison of tumour coverage and dose to OAR between the proton and photon plans. Figure 4B illustrates robust evaluation for the proton plan with the aforementioned uncertainty scenarios where small volume OARs show large variation with the given uncertainty scenarios as expected. A comparison of tumour coverage and dose to OAR between the proton and photon plans is summarised in Table 2.

**Table 2:** Dose‐volume metrics scores for Case 2.

Structure	Dose‐volume metrics	Objectives	Proton‐IMPT	Photon‐VMAT	Difference (Gy) (IMPT‐VMAT)
PTV_5040	D98%	≥47.88 Gy	48.4^a^	48.9^a^	−0.5^a^
CTV_5040	D98%	≥47.88 Gy	48.5	49.7	−1.2
Brainstem	D0.03cc	<52.9 Gy	44.3	37.7	6.6
Optic_Chiasm	D0.03cc	<52.9 Gy	51.3	50.9	0.4
OpticNrv_L	D0.03cc	<52.9 Gy	27.3	14.4	12.9
OpticNrv_R	D0.03cc	<52.9 Gy	39.8	32.9	6.9
Pituitary	Mean Gy	<40.0 Gy	44.0	36.8	7.2
Hypothalamus	Mean Gy	<40.0 Gy	40.9	35.5	5.4
Cochlea_L	Mean Gy	<35.0 Gy	0.0	1.6	−1.6
Cochlea_R	Mean Gy	<35.0 Gy	0.0	2.2	−2.2
Lobe_Temporal_L	Mean Gy	<10.0 Gy	3.5	3.0	0.5
Lobe_Temporal_R	Mean Gy	<10.0 Gy	3.4	4.2	−0.8
Hippocampus_L	Mean Gy	<14.0 Gy	2.3	5.7	−3.4
Hippocampus_R	Mean Gy	<14.0 Gy	40.9	7.8	33.1
Gradient Index^b^	(= V50%/V100%)		35.72	7.13	
Homogeneity Index^c^	(= D2%/D98%)		1.06	1.05	
Integral dose	Gy cc		4597.46	6105.2	

L, left; OpticNrv, optic nerve; R, right.

^a^ PTV coverage for the Proton plan is not applicable as CTV‐based robust optimisation is implemented but displayed for illustration purposes only. VMAT PTV D99% >95% of prescription dose ensures excellent robustness against setup error, which is confirmed by ExacTrac IGRT with 0.5 mm/0.5° action limit.

^b^ Gradient index is defined by the ratio of V50% to V100% of prescription dose.^7^

^c^ Homogeneity Index is defined by the ratio of near‐max (D2%) to near‐min (D98%).^8^

The photon plan and the proton plan had comparable doses to target volumes, both cochlea and temporal lobes (Table 2). However, the proton plan had higher doses to the optic chiasm, both optic nerves, pituitary gland and hypothalamus. The gradient index was higher and integral dose was reduced by 25% in the proton plan. The V_5Gy_ for the whole brain was higher by 1.4% in the proton plan. After careful evaluation of the dosimetry and further discussion within the MDT the decision was made to proceed with photon irradiation and treatment was well tolerated.

## Discussion

Advances in planning and treatment delivery techniques have allowed photon irradiation to become more conformal and lower doses to normal tissues. This is particularly important for paediatric and AYA cancer patients to minimise the risk of late effects. Inherent physical differences mean that PBT can further decrease the dose to normal tissues. However, the dose to OARs immediately adjacent to tumour volumes can be higher in PBT due to beam properties and margins required for setup and range uncertainties. Some proton facilities use beam‐modifying devices to reduce the lateral penumbra to minimise this. Both cases presented in this article had reduced doses of OARs adjacent to tumour volumes in the photon plan compared to the proton plan. Photon irradiation can achieve sharp dose fall‐off adjacent to OARs, which is even more pronounced when using the adapted stereotactic approach for small tumours as described in this report.

Case 1 highlights the complexity of treating very young paediatric patients with brain tumours. Multiple factors such as the patient's age, diagnosis, tumour size and tumour location play a crucial role in deciding on a suitable treatment plan. The standard treatment for paediatric patients with intracranial ependymomas of WHO grade 2/3 is maximal safe resection followed by local radiation therapy.^9^ Non‐randomised PBT studies for localised paediatric ependymoma have shown comparable disease outcomes to photon irradiation.^10^, ^11^, ^12^ In the largest series to date reporting the long‐term outcomes of PBT for 386 paediatric patients with ependymoma, the 7‐year local control and overall survival rates were 77% and 82.2%, respectively.^10^ Currently, there is no randomised evidence comparing PBT with photon irradiation for paediatric ependymomas.

Photon irradiation was recommended for Case 1 instead of PBT due to the lower dose to the hippocampi which were adjacent to the tumour volume. The mean dose to the left hippocampus in the photon plan was 20.8 Gy versus 36.6 Gy. The mean dose to the right hippocampus was 12.2 Gy versus 34.8 Gy. The temporal lobe mean doses were <10 Gy in both plans, however, the left temporal dose was slightly higher with protons. There is limited evidence on the dose constraints for the hippocampi and temporal lobes in paediatric patients. Jalali et al. conducted a prospective trial of 48 patients with benign/low‐grade brain tumours treated with stereotactically guided conformal radiotherapy.^13^ A mean left hippocampus dose of 30 Gy predicted for 10% change in long‐term full‐scale IQ and performance IQ using the Wechsler‐Bellevue Intelligence Scale. Interestingly there was no relationship between right hippocampus dose and neurocognitive outcome. A 10‐year neurocognitive longitudinal study of paediatric and AYA low‐grade glioma survivors found that children <12 years old with greater hippocampus dose were associated with a greater memory decline.^14^ The significance of reduced low dose to normal brain using PBT is to be determined. Currently there is an open international trial comparing neurocognitive change and functional outcomes in paediatric brain tumour patients treated with photons versus protons.^15^

The dose to the pituitary gland in Case 1 was moderately higher with photons than protons (17.9 Gy vs. 15.5 Gy) and the risk of endocrine dysfunction is similar for both dose levels. Managing endocrine dysfunction is not always easy and usually requires hormone replacement.^16^ Unfortunately, there are limited therapeutic interventions to manage neurocognitive dysfunction.

For Case 2, the mean pituitary dose with photons was lower than protons (36.8 Gy vs. 44 Gy). The dose to the whole brain (V_5Gy_), brainstem, optic chiasm, optic nerves, hippocampi and temporal lobes was also lower (Table 2).

Optic pathway gliomas are low‐grade tumours that when treated have a very good prognosis with an overall survival at 10 years of over 90%.^17^ Hence reducing the dose to normal tissues is important to minimise late effects. For these reasons, PBT has been used to treat young patients with low‐grade gliomas with reports of good local control and overall survival.^18^ However, this case illustrates that there remains a role for stereotactic photon irradiation for optic pathway gliomas. Furthermore, there is an increasing number of studies exploring the use of photon stereotactic radiosurgery (SRS) for paediatric patients with brain cancer. Ge et al. reported the outcomes of 52 patients, aged 2–53 years, with optic pathway glioma treated by a single fraction or fractionated (2–4 fractions) Gamma Knife SRS showing good local control and visual acuity preservation.^19^ A recent dosimetric study also showed the advantages of using mask‐based Gamma Knife fractionated stereotactic radiotherapy compared to photon VMAT for adult and paediatric brain tumours.^20^ Another treatment option might be proton SRS, which has been shown to be safe and effective for different intracranial pathologies.^21^

Both cases had a reduction in the integral dose using PBT (35% and 25%). The lower dose to normal tissues using PBT is expected to reduce the risk of secondary malignancies and other toxicities. However, the effect of neutron scatter from PBT remains unanswered. Although radiation‐induced secondary malignancies are rare, they can worsen a patient's quality of life and reduce life expectancy. Modelling studies and early clinical data have suggested a lower risk after PBT but larger studies with long follow‐up are needed.^22^ A large multicentre Paediatric Proton and Photon therapy Comparison Cohort study is under development to compare the risk of subsequent cancers after PBT with photons including the dose‐volume effects.^23^

These two cases have highlighted how dosimetric differences between PBT and photons can help decide on the treatment modality. Multiple factors are also considered. Critically there is no proton facility in Australia, and travelling overseas for PBT can be a difficult and stressful process.^24^ Even with government funding, some patients and families decide not to go overseas. Developing proton facilities locally will help improve access to PBT.

## Conclusion

This case report highlights the need for a tailored approach when deciding whether a patient should be treated with photon irradiation or PBT. We suggest that PBT does not always result in superior radiation plans compared to photon irradiation for paediatric and AYA patients. Future studies including comparative treatment planning may help identify other factors in addition to age and diagnosis that would guide treatment selection.

## Conflict of Interest

The authors declare no conflict of interest.

## Ethics Statement

Case reports determined not to require Ethics Committee review as per our institutional policy.

## Patient Consent

Written informed consent for publication was obtained.

## Data Availability

The data that support the findings of this study are available from the corresponding author upon reasonable request. The data are not publicly available due to privacy or ethical restrictions.
